# Elastic and Electronically Inelastic Cross Sections for the Scattering of Electrons by Pyrrole

**DOI:** 10.1021/acs.jpca.4c02719

**Published:** 2024-06-11

**Authors:** Murilo O. Silva, Romarly F. da Costa, Márcio H. F. Bettega

**Affiliations:** † Instituto Federal do Paraná, Campus Avançado Goioerê, Rodovia Luiz Dechiche, s/no, 87360-000 Goioerê, Paraná, Brazil; ‡ Departamento de Física, Universidade Federal do Paraná, Caixa Postal 19044, 81531-980 Curitiba, Paraná, Brazil; § Centro de Ciências Naturais e Humanas, Universidade Federal do ABC, 09210-580 Santo André, São Paulo, Brazil

## Abstract

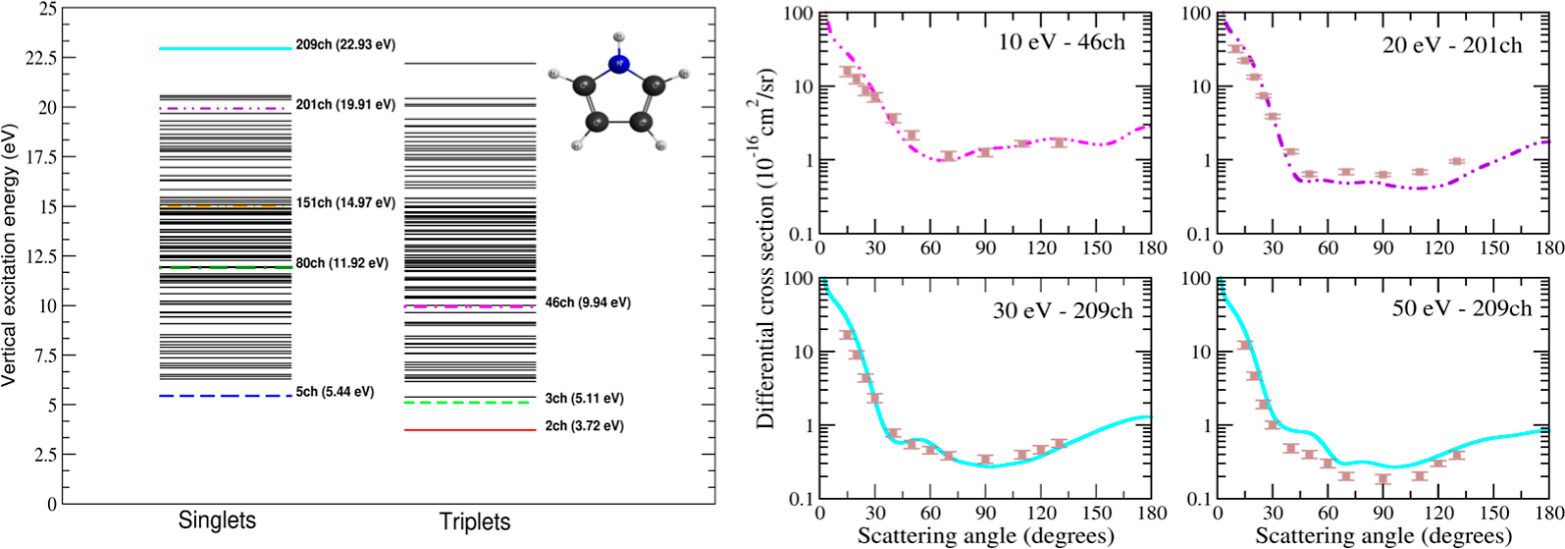

Integral and differential cross sections for elastic and electronically inelastic electron scattering from the pyrrole molecule are reported. The cross section calculations employed the Schwinger multichannel method with norm-conserving pseudopotentials. The collision dynamics was described according to a model in which up to 209 energetically accessible channels were treated as open. In the elastic channel, calculations carried out in the interval of energies from 0 to 50 eV revealed the presence of four resonances with peaks located at 2.56 eV (π_1_^*^), 3.82 eV (π_2_^*^), 4.70 eV (σ_NH_^*^), and between 8.30 and 9.50 eV (σ*) positions which are in good agreement with previous assignments. Moreover, the role of the multichannel coupling effects in obtaining accurate cross sections was evaluated by comparing the present results with theoretical results recently reported in the literature and early measurements performed for elastic electron collisions with furan. Electronic excitation cross sections involving the transitions from ground state to the 1^3^B_2_, 1^3^A_1_, 1^1^A_2_, and 1^1^A_1_ excited states of pyrrole driven by electron impact are presented for energies from thresholds up to 50 eV and, whenever possible, critically compared with the data available in the literature.

## 1 Introduction

Scattering of low-energy electrons by molecules of biological relevance has received increasing attention since the study conducted by Boudaïffa et al.^1^ These authors identified that the genotoxic effects resulting from exposure of living cells to ionizing radiation are not exclusively attributed to the direct action due to the impact of high-energy primary photons. On the contrary, potential lesions induced in DNA, whether lethal or not, also originate from secondary species generated by the primary beam of ionizing radiation. Among these secondary species, low-energy electrons with energies typically ranging between 1 and 20 eV are the most abundant.^2^ Motivated by this context, many theoretical and experimental research groups seek to contribute to the full understanding of electron damage to DNA by conducting studies of electron interactions with precursor molecules such as nucleobases,^3−6^ sugar structures,^7^ amino acids,^8^ phosphates,^9−11^ and also with molecules that can be considered as simple prototypes for larger systems.^12^

Five-membered heterocyclic systems play a crucial role in both chemistry and biology, being indispensable for the existence and maintenance of life. These ring molecules, composed of carbon atoms and heteroatoms such as nitrogen, oxygen, or sulfur, are widely recognized for their versatility and significance in various fields of basic and applied research, particularly in the pharmaceutical industry.^13,14^ Many complex biological systems (including vitamins, hemoglobin, hormones, and others) feature these heterocyclic rings as basic structural components.^15^ Furthermore, the presence of these molecules in a compound provides greater metabolic stability, solubility, and bioavailability, characteristics that are crucial in drug design and development. Due to their unique physicochemical and biological properties, five-membered heterocycles are therefore considered as key structural elements in the composition of a wide variety of medications.^13^

**Figure 1:**
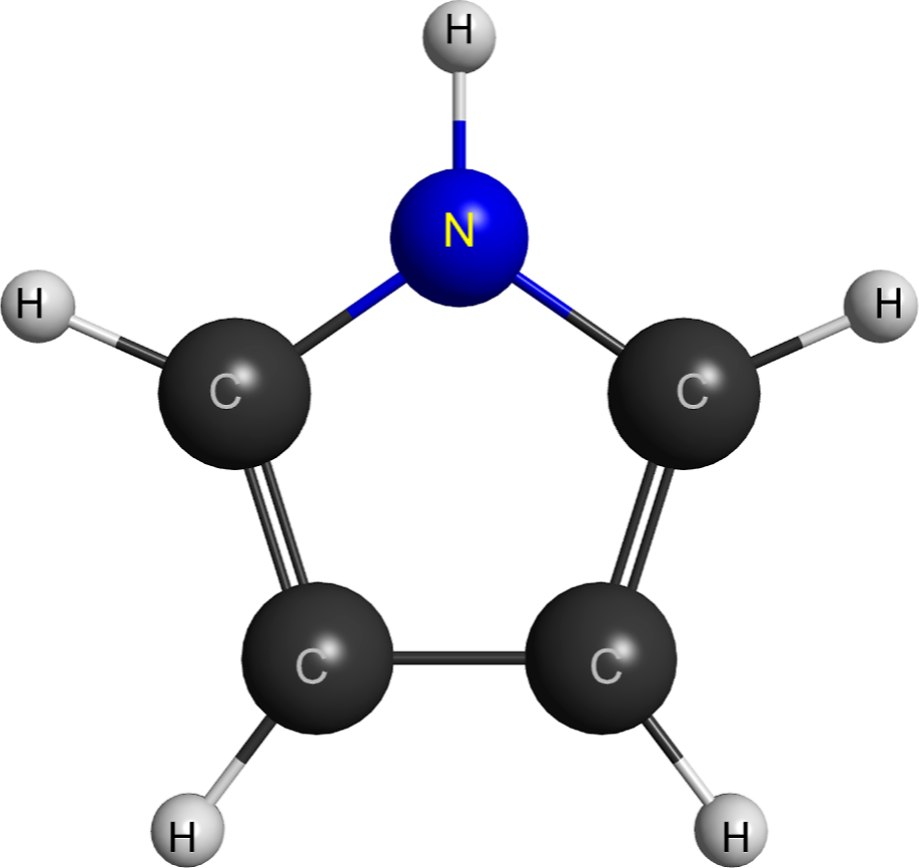
Ball and stick model of the pyrrole molecule generated with MacMolPlt.^35^

In this work, we focus on the pyrrole (C_4_H_4_NH) molecule, where in Figure 1 the molecular structure is presented. Besides serving as a simple model of the basic constituents of DNA, such as purine, pyrrole is a relevant organic system in medicinal chemistry, especially in the context of developing new synthetic reactions and pharmaceutical products. In addition, this system possesses anticancer, antimicrobial, and antiviral activities.^16^ Finally, a literature survey suggests that polyamides containing *N*-methylimidazole and *N*-methylpyrrole amino acids can form complexes in the minor groove of DNA (the region where the distance between the DNA nitrogenous bases is smaller).^17^ This allows for the specificity of the DNA sequence to be controlled by the order of the pyrrole and imidazole amino acids, implying that these molecules can bind to specific base pairs in the DNA chains.

Regarding the study of electron interactions with the pyrrole molecule, Van Veen^18^ was a pioneer in identifying and characterizing two resonant structures through the use of the electron transmission spectroscopy (ETS) and low-energy electron-impact spectroscopy techniques. In the ETS measurements, the resonances were centered at 2.38 and 3.44 eV, while in the results obtained by electron impact technique, the resonances were located at 2.50 and 3.60 eV. Subsequently, Modelli and Burrow^19^ also employed the ETS technique to identify and characterize nine organic compounds, including pyrrole, and reported the presence of two π* resonant structures for pyrrole peaked at 2.36 and 3.45 eV. These findings were further confirmed by the ETS measurements performed by Pshenichnyuk et al.,^20^ in which the occurrence of the centers of the resonances was reported at 2.33 and 3.44 eV. In a study combining theoretical and experimental approaches, Mukherjee et al.^21^ also identified the presence of two resonances. In the theoretical results obtained by these authors, the resonances were centered at 2.63 and 2.92 eV (π_1_^*^) and 3.27 and 3.53 eV (π_2_^*^), while in the experimental measurements using two-dimensional electron energy loss spectra (EELS), they were observed at 2.50 and 3.50 eV, respectively. From the theoretical side, by considering only the elastic channel in their calculations carried out according to the Schwinger multichannel method, de Oliveira et al.^22^ identified four resonances, two of them of π* character, with centers at 2.70 and 3.80 eV, while the other two of σ* character, centered at 4.00 and 8.70 eV. More recently, through the R-matrix method at the CAS-CI level of approximation to consider both elastic and inelastic channels, Tomer et al.^23^ reported three resonances in the elastic channel. For calculations carried out with the STO-3G basis set, the resonances were centered at 1.30, and 3.30 eV and in the energy range between 8.00 and 10.00 eV, whereas for the DZP basis set, the resonances were centered at 3.60 and 4.70 eV.

In the present study, we report on differential and integral cross sections (ICSs) for elastic and electronically inelastic scattering of low-energy electrons by pyrrole, covering energies up to 50 eV. We employed the Schwinger multichannel (SMC) method implemented with pseudopotentials^24^ to obtain the scattering amplitudes and have incorporated the effects of channel coupling (ranging from 1 open channel to 209 open channels) by using the minimal orbital basis for single configuration interactions (MOB-SCI)^25^ strategy. In addition to describing the resonant states, our goal was to assess the influence of the multichannel coupling effects up on the elastic channel and to investigate electronic excitation processes involving transitions from the ground state to the 1^3^B_2_, 1^3^A_1_, 1^1^A_2_, and 1^1^A_1_ electronically excited states of pyrrole driven by electron impact, to be compared with the results obtained by Tomer et al.^23^ using the R-matrix method.

The remainder of this paper is structured as follows: theoretical aspects are provided in Section 2, and computational details of the calculations are addressed in Section 3. In Section 4, we present and critically analyze the cross-section results obtained through the SMC method, comparing them with previous calculations and measurements. Finally, the conclusions are summarized in Section 5.

## 2 Theory

The elastic and electronically inelastic cross sections presented in this work were obtained using the SMC method^26,27^ implemented with the norm-conserving pseudopotentials proposed by Bachelet, Hamann, and Schlüter (BHS).^28^ The SMC method is a variational approach to the scattering amplitude in which important effects that occur during electron–molecule collisions, such as exchange, target polarization, and multichannel coupling are considered. Since the SMC method has been reviewed in ref (29), here we will present only the aspects of this method that are pertinent to the present calculations. In the SMC method, the resulting expression for the scattering amplitude is as follows
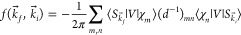
1where
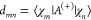
2and the operator A^(+)^ is given by
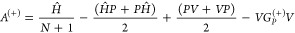
3

In the above equations,  is an eigenstate of the unperturbed Hamiltonian *H*_0_ = *H*_*N*_ + *T*_*N*+1_ and is given by the product of a target state and a plane wave with  representing the momentum of the free incident (scattered) electron. In the definition of *H*_0_, *H*_*N*_ represents the target Hamiltonian, and *T*_*N*+1_ corresponds to the kinetic energy operator of the incident electron; *V* is the interaction potential between the incident electron and the target’s electrons and nuclei; *Ĥ* = *E* – *H*, where *E* is the total collision energy and *H* is the (*N* + 1)-electron Hamiltonian in the fixed nuclei approximation; *G*_*P*_^(+)^ = *PG*_0_^(+)^ is the free-particle Green’s function projected into the *P*-space, and *P* is a projection operator onto the open-channel space of the target, which is given by
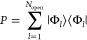
4where |Φ_*l*_⟩ are target states that can be either the ground state or some electronically excited state of the *N*-electron molecular target and, finally, *N*_open_ is the number of open channels, that is, target states that become energetically accessible to the electron–molecule system as the energy of the incident electron increases during the collision process. The |χ_*mn*_⟩ represents a basis set of (*N* + 1)-electron Slater determinants (CSFs—configuration state functions), which are constructed as spin-adapted products of target states with single-particle scattering orbitals and are given by
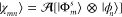
5where  is the antisymmetrization operator, |Φ_*m*_^*s*^⟩ denotes an *N*-electron Slater determinant obtained through single excitations of the target from the valence occupied (hole) orbitals of the ground (reference) state to a set of unoccupied (particle) orbitals with spin *s* (*s* = 0 for singlet or *s* = 1 for triplet states), where *m* = 1 corresponds to the ground state and |ϕ_*n*_⟩ is a scattering orbital.

## 3 Computational Details

The ground state geometry of pyrrole was optimized in the *C*_2*v*_ point group through the second-order Møller–Plesset perturbation theory (MP2) with the aug-cc-pVDZ basis set by using GAMESS^30^ computational package. The nuclei and core electrons of carbon and nitrogen atoms are replaced by the pseudopotentials of BHS, while the valence electrons are described with a set of 5s5p2d uncontracted Cartesian Gaussian (CG) functions generated according to the procedure described in ref^31^. The exponents of these CG functions are listed in Table 1. To describe the hydrogen atoms, we employed the 4s/3s basis set of Dunning^32^ increased by one p-type function with the exponent equal to 0.75. The target ground state was described at the Hartree–Fock level, while the excited states were obtained according to the minimal orbital basis for the single configuration interaction (MOB-SCI) strategy.

**Table 1:** Exponents of the Uncontracted Cartesian Gaussian Functions Used for Carbon (C) and Nitrogen (N) Atoms in the Present Calculations Performed with the SMC Method

type	C	N
s	12.49628	17.56734
s	2.470286	3.423615
s	0.614028	0.884301
s	0.184028	0.259045
s	0.039982	0.055708
p	5.228869	7.050692
p	1.592058	1.910543
p	0.568612	0.579261
p	0.210326	0.165395
p	0.072250	0.037192
d	0.603592	0.403039
d	0.156753	0.091192

The bound state and scattering calculations employed improved virtual orbitals (IVOs)^33^ to represent particle and scattering orbitals. Through a full single configuration interaction (FSCI) calculation, a total of 4602 excited states (associated with 2301 hole–particle pairs), being 2301 singlets and 2301 triplets, were obtained. We then selected from these 4602 states the lowest 80 excited states (the states of interest), corresponding to 10 singlet and 10 triplet states from each irreducible representation of the *C*_2*v*_ group. From the 2301 hole–particle pairs of the FSCI calculation, we chose 104 hole–particle pairs to describe these 80 states of interest through the MOB-SCI strategy, ensuring that the energies associated with these excited states were described within 90% agreement with the FSCI results.

**Figure 2:**
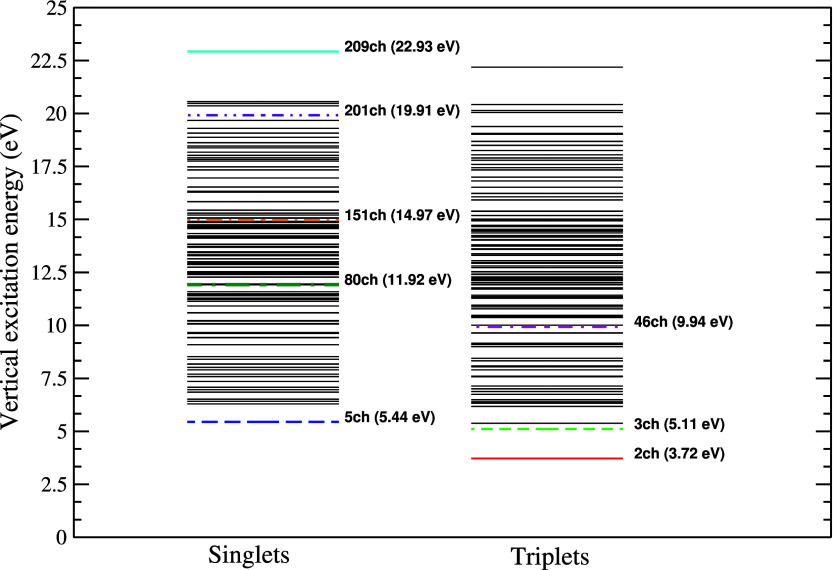
Schematic representation of the vertical excitation energies (in eV) of the 208 electronically excited states of pyrrole obtained with the MOB-SCI strategy and the different multichannel coupling schemes employed in the present scattering calculations performed by means of the SMC method.

We present in Tables 2 and 3 the vertical excitation energies for the first four electronically excited triplet states and the first 14 electronically excited singlet states of the pyrrole molecule. The energy spectra obtained with MOB-SCI and FSCI (which is taken as our reference) strategies are in excellent agreement with each other. Comparison of MOB-SCI energies with theoretical^36−39^ and experimental^18,36^ results shows fair agreement for the low-lying states and reasonable agreement for higher energy states. There is also an inversion in the order of some excited states obtained in the FSCI and MOB-SCI calculations if we compare this with the results from more robust electronic structure calculations. In Figure 2, we provide a schematic representation of the excitation energy spectrum obtained with the MOB-SCI strategy. The color lines represent different channel-coupling schemes used in our calculations.

**Table 2:** Vertical Excitation Energies (in eV) for the First Four Electronic Excited Triplet States Obtained from Present FSCI and MOB-SCI Calculations^a^

state	FSCI	MOB-SCI	(36)	(37)	(38)	exp.^18^
1^3^B_2_	3.46	3.72	4.58	4.65	4.97	4.20
1^3^A_1_	4.92	5.11	5.60	5.84	6.20	5.10
1^3^A_2_	5.29	5.37	5.08	5.17	11.00	
1^3^B_1_	6.10	6.19	5.82	5.82	10.27	

a We compared our results with the theoretical results available in the literature obtained by Wan et al.^36^ using the symmetry adapted cluster–configuration interaction (SAC–CI) method by Nakatsuji et al.^37^ using the SAC–CI method by Palmer et al.^38^ using the multi-reference multi-root CI method and the experimental data obtained by Van Veen^18^ using the EELS technique.

**Table 3:** Vertical Excitation Energies (in eV) for the First 14 Excited Electronic Singlet States Obtained from FSCI and MOB-SCI Calculations^a^

state	FSCI	MOB-SCI	(36)	(37)	(38)	(39)	exp. (listed from ref (36))
1^1^A_2_	5.38	5.44	5.11	5.20	5.03	5.20	5.22
1^1^B_1_	6.24	6.29	5.80	5.85	5.68	5.95	5.70
2^1^A_2_	6.33	6.41	5.81	5.95	5.71	5.94	
1^1^B_2_	6.35	6.51	5.88	5.97	5.86	6.04	5.86
2^1^B_1_	6.72	6.84	6.05	6.13	5.77	6.12	
2^1^B_2_	6.79	7.35	6.48	7.52	6.48	6.57	6.20–6.50
3^1^B_1_	6.89	6.96	6.39	6.70	6.27	6.55	6.42
3^1^A_2_	7.02	7.07	6.38	6.85	6.25	6.51	
2^1^A_1_	7.32	7.58	6.41	6.68	6.66	6.37	
4^1^B_1_	7.59	7.69	6.68		6.43	6.82	6.50–6.70
4^1^A_2_	7.62	7.89	6.44		6.37	6.57	
3^1^A_1_	7.96	8.01	6.64	6.89	6.54	6.87	
5^1^A_2_	7.98	8.17	6.71		6.92		
3^1^B_2_	9.09	9.62	6.76	7.59	6.71	6.90	6.78

a We compared our results with the theoretical results available in the literature obtained by Wan et al.^36^ using the symmetry adapted cluster–configuration interaction (SAC–CI) method by Nakatsuji et al.^37^ using the SAC–CI method by Palmer et al.^38^ using the multi-reference multi-root CI method by Nakano et al.^39^ using the complete active space self-consistent field (CASSCF) method and the experimental data listed in the work of Wan et al.^36^

The same 104 hole–particle pairs used to build the active space in the MOB-SCI strategy were also employed in the construction of the CSF space used to represent the target polarization. The number of CSFs obtained for each irreducible representation is 10,097 for A_1_, 7605 for A_2_, 10,016 for B_1_, and 7678 for B_2_. To label the different channel-coupling schemes, we used the acronym *N*_open_ch-SEP,^34^ where *N*_open_ is the number of open channels as mentioned before. For example, if *N*_open_ is equal to 1, only the elastic channel is open; if *N*_open_ is equal to 2, both the elastic and the first inelastic channels are open, and so on. Regarding the level of channel coupling, the scattering calculations were conducted according to 2ch, 3ch, 5ch, 46ch, 80ch, 151ch, 201ch, and 209ch levels of approximation, where the thresholds for each calculation level are presented by the color lines in Figure 2.

Pyrrole is a polar molecule, with a calculated permanent dipole moment of 1.87 D in good agreement with the experimental value of 1.84 D.^40^ Additionally, the calculated polarizability also demonstrates excellent agreement with the experimental value, yielding a computed value of 7.84 Å^3^, consistent with the experimental result of 7.94 Å^3^.^41^ Therefore, considering the long-range potential caused by the dipole moment is a necessary step to a full description of the collision process, especially at lower scattering angles. However, in the SMC method, square-integrable functions (*L*^2^) are used to expand the scattering wave function, which truncates the long-range interaction of the dipole. To address this issue, we included the long-range interaction in the cross sections using the Born-close procedure for the scattering amplitude following the same strategy described in ref (29). The Born-closure procedure combines the scattering amplitude from the SMC method (lower partial waves) with the scattering amplitude of the dipole potential (higher partial waves) calculated in the first Born approximation (FBA). Briefly, we expand the scattering amplitude obtained with the SMC method in partial waves up to a certain value of *l*_SMC_, and we expand the scattering amplitude of the dipole potential calculated in the FBA from *l*_SMC_ + 1 to ∞. The values of *l*_SMC_ are selected by comparing the DCSs obtained with and without the Born-closure procedure, which coincide above approximately 20°, and they depend on the energy of the incident electron. In the calculations performed in this work, the *l*_SMC_ values were chosen as follows: *l* = 1 from 0.1 to 0.9 eV; *l* = 2 from 1 to 2 eV; *l* = 3 from 2.1 to 3 eV; *l* = 4 from 3.1 to 4.4 eV; *l* = 5 from 4.5 to 5.4 eV; *l* = 6 from 5.5 to 6.5 eV; *l* = 7 from 7 to 8 eV; *l* = 8 from 8.5 to 8.9 eV; *l* = 9 from 9 to 9.5 eV; and *l* = 10 from 10 to 50 eV.

## 4 Results and Discussion

**Figure 3:**
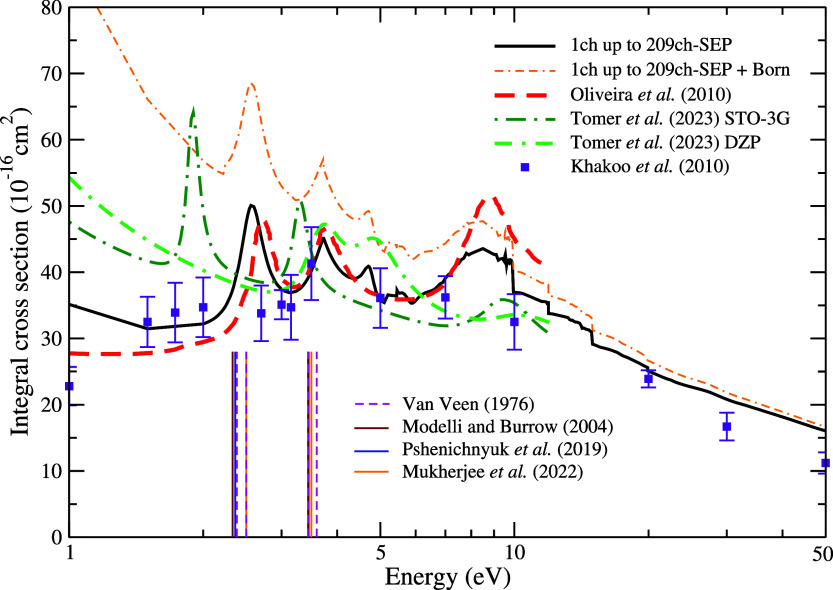
Integral cross section (ICS) for elastic electron scattering by pyrrole. Solid black line presents SMC results considering all channels energetically accessible to the molecular target up to 50 eV without the Born-closure procedure; double-dashed-dotted orange line presents SMC results considering all channels energetically accessible to the molecular target up to 50 eV with the Born-closure procedure; dashed red line presents results obtained by de Oliveira et al.^22^ using the SMCPP method; dashed-dotted dark green and double-dashed-dotted light green lines present results obtained by Tomer et al.^23^ using the R-matrix method with the STO-3G and DZP basis set, respectively; violet squares present measured data for elastic electron scattering by the furan molecule obtained by Khakoo et al.^42^ The vertical bars indicate the experimental resonances positions: dashed magenta line, data obtained by Van Veen;^18^ solid dark red line, data obtained by Modelli and Burrow;^19^ solid blue line, data obtained by Pshenichnyuk et al.;^20^ and solid orange line, data obtained by Mukherjee et al.^21^ See the text for further discussion.

In Figure 3, we present the ICS for electron scattering by the pyrrole molecule obtained with the SMC method, for impact energies ranging from 1 to 50 eV. The vertical bars indicate the experimental resonance positions. We show the results at the static-exchange plus polarization approximation with the inclusion of the multichannel coupling effects by considering calculations from 1ch-SEP up to 209ch-SEP with and without the Born-closure procedure. At the present level of calculation, four resonant structures were observed, being located at 2.56 (π_1_^*^), 3.72 (π_2_^*^), 4.70 (σ_NH_^*^), and between 8.30 and 9.50 eV (σ*). In the 1ch-SEP up to 209ch-SEP calculation, the second resonance is affected by the presence of an upcoming excited state since it is located right at the threshold of the first triplet excited state. The characters and positions of the four resonances identified in our calculations are in agreement with the results found by de Oliveira et al.^22^ and by Tomer et al.^23^ except by the fact that the last authors only identified the occurrence of three resonances. Regarding the positions of the resonances listed in Table 4, the first resonance obtained in our work is 0.14 eV below while our second resonance is 0.02 eV above the position of that obtained by de Oliveira et al.,^22^ who considered only the elastic channel as open. The third resonance identified in our calculations, attributed as a resonance of σ_NH_^*^ character by de Oliveira et al.,^22^ is located 0.7 eV above the one obtained by these authors. We suspect that this resonance may have a mixed shape and core-excited nature as we have identified a structure in the same energy range in the excitation cross section from the ground state to the first triplet state, as will be discussed below. As for the higher lying resonance, we observed that it falls within the energy range as those reported by the de Oliveira et al.^22^ As mentioned before, the results obtained by Tomer et al.^23^ using the R-matrix method display the presence of three resonances. In the work developed by these authors, the excited states were described using the CAS-CI method with the use of the STO-3G and DZP basis sets. Both calculations display two shape resonances. In the calculation with the STO-3G basis, these resonances occur at 1.90 and 3.30 eV, while in the calculation with the DZP basis, they are centered at 3.60 and 4.70 eV. The discrepancy in the positions of the resonances is attributed by the authors to the size of the configuration space used in each calculation. Only the second resonance calculated by Tomer and co-workers with the use of the STO-3G basis set is consistent with the position of the resonance obtained in our calculations. Additionally, the authors identified a broader structure between 8 and 10 eV in the calculation using the STO-3G basis set, claimed by them to be in good agreement with the results presented by de Oliveira et al.^22^ Our results also corroborate the presence of this structure. Van Veen^18^ identified two resonant structures through the use of transmission spectrum (centered at 2.38 and 3.44 eV) and electron impact (centered at 2.50 and 3.60 eV) techniques. The values from the transmission spectrum are in good agreement with our results, while the values from electron impact experiments show only reasonable agreement. Additionally, Modelli and Burrow^19^ identified and characterized resonances at 2.36 and 3.45 eV using the ETS technique, while more recent measurements conducted by Pshenichnyuk et al.,^20^ also using ETS, detected resonances at 2.33 and 3.44 eV and also identified a resonance at 0.50 eV. Furthermore, Mukherjee et al.,^21^ in a combined theoretical (involving electronic structure calculations) and experimental (using EELS) study, identified the same resonances previously reported in the literature. Table 4 summarizes the theoretical results (upper part) and experimental results (lower part) for the resonance positions. The first theoretical position presented in the work by Mukherjee et al.^21^ listed in Table 4 deals with adiabatic and vertical positions. The present results are in good agreement with the resonance positions concerning both theoretical and experimental assignments, especially for the first resonance and with reasonable agreement for the second one. For the magnitude of the present ICS, as expected, when considering all energetically accessible channels (from 1 to 209 channels open up to 50 eV), we obtained a smoother curve. This happens because, in a conventional calculation (which considers only the elastic channel as accessible), there are gaps for pseudoresonances that arise from channels that although should be open, are treated as closed in the elastic channel. Allowing the opening of these channels in the calculations results in a more uniform cross section. Furthermore, there is a reduction in the magnitude of the ICS due to flux competition between the elastic and electronically inelastic channels. In the 1ch-SEP up to 209ch-SEP calculation, at an energy of 10 eV, there is an abrupt drop in the magnitude of the ICS, which is consistent with a significant steal of flux in this region due to the increasing number of open channels, going from the 5ch-SEP calculation to the 46ch-SEP calculation. At lower energies, there is an increase in the ICS with Born-closure, which is a characteristic of scattering by polar molecules. Due to the lack of experimental data for electron scattering cross section of the pyrrole molecule, we compared our results with the elastic electron scattering data for the furan molecule, as obtained by Khakoo et al.^42^ The interest in this comparison arises from the fact that both pyrrole and furan are heterocyclic organic compounds containing a five-membered ring with carbon and either nitrogen or oxygen atoms, respectively. The cross sections for both systems have similar magnitudes and behavior. At low energies, a difference in the ICS of the two systems is observed due to the long-range interaction caused by the permanent dipole moment of the polar systems. The dipole moment of the pyrrole molecule is considerably higher than that of the furan molecule (1.87 and 0.68 D,^42^ respectively), which explains the observed difference. For energies less than 30 eV, our results fall within the error margins of the experimental data.

**Table 4:** Comparison between the Positions of the Resonances Observed in the Elastic Scattering of Electrons by the Pyrrole Molecule

calculation level	π_1_^*^	π_2_^*^	σ_NH_^*^	σ_ring_^*^
present SMC results	2.56	3.82	4.70	8.30–9.50
de Oliveira et al.^22^	2.70	3.80	4.00	8.70
Tomer et al.^23^	1.90 (STO-3G), 3.60 (DZP)	3.30 (STO-3G),4.70 (DZP)		8.00–10.00
Mukherjee et al.^21^	2.63 and 2.92	3.27 and 3.53		

experimental results	π_1_^*^	π_2_^*^
Modelli and Burrow^19^	2.36	3.45
Van Veen^18^	2.38 and 2.50	3.44 and 3.60
Pshenichnyuk et al.^20^	2.33	3.44
Mukherjee et al.^21^	2.50	3.50

**Figure 4:**
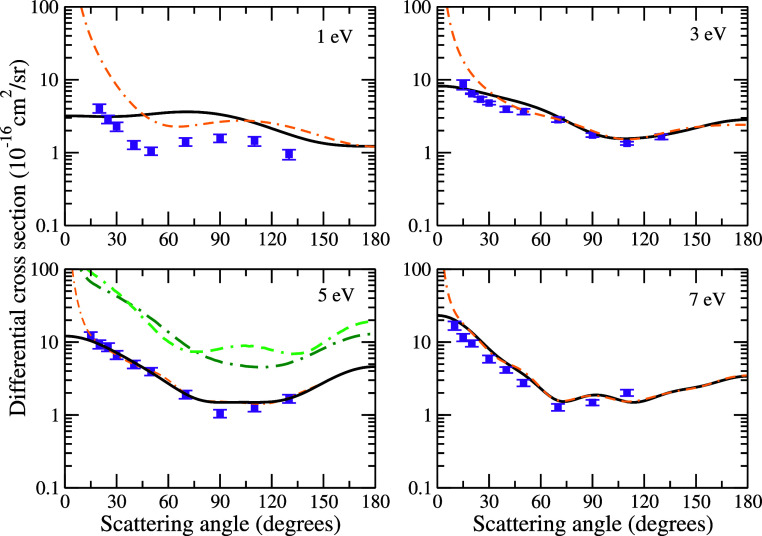
Differential cross sections for elastic electron scattering by pyrrole at the impact energies of 1, 3, 5, and 7 eV. Solid black line and double-dashed-dotted orange line present SMCPP results obtained according to the best multichannel coupling scheme (1ch at 1 eV, 1ch at 3 eV, 2ch at 5 eV, and 5ch at 7 eV) without and with Born-closure procedure, consecutively; dashed-dotted dark green and double-dashed-dotted light green lines present results obtained by Tomer et al.^23^ using the R-matrix method with the STO-3G and DZP basis set, respectively; and violet squares present measured data for elastic electron scattering by the furan molecule obtained by Khakoo et al.^42^ See the text for further discussion.

**Figure 5:**
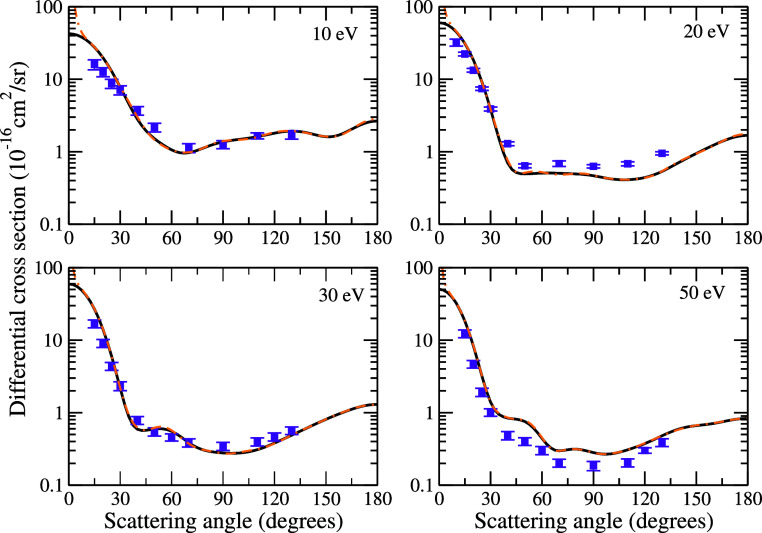
Differential cross sections for elastic electron scattering by pyrrole at the impact energies of 10, 20, 30, and 50 eV. Solid black line and double-dashed-dotted orange line present SMCPP results obtained according to the best multichannel coupling scheme (46ch at 10 eV, 201ch at 20 eV, and 209ch at 30 and 50 eV) without and with Born-closure procedure, respectively; and violet squares present measured data for electron scattering by the furan molecule obtained by Khakoo et al.^42^ See the text for further discussion.

The DCSs for the elastic channel are presented in Figures 4 and 5 at 1, 3, 5, 7, 10, 20, 30, and 50 eV. We show each DCS at the best level of channel-coupling with and without the inclusion of the Born-closure procedure. Our results are compared with theoretical results obtained by Tomer et al.^23^ for the only available energy of 5 eV and with the previous SMC results reported by de Oliveira et al.^22^ Also included for comparison is the experimental data for the furan molecule obtained by Khakoo et al.^42^ A big discrepancy between the magnitude of the SMC and R-matrix DCSs at 5 eV is observed. On the other hand, our result is consistent with the results of de Oliveira et al.^22^ for pyrrole and with the measurements of Khakoo et al.^42^ for furan. Still regarding the comparison with the experimental data, some differences are observed at energies of 1 and 3 eV, especially for scattering angles below 50° at 3 eV. This suggests that the de Broglie wavelength of the incident electron is larger than the molecular structure, allowing the electron to distinguish the differences between the details of the molecular targets. For higher energies, we observed a good agreement between the experimental data and our results, particularly for energies above 10 eV, where the de Broglie wavelength of the incident electron becomes comparable to the size of the molecular structure of the two systems, pyrrole and furan, which may hinder distinguishing between the structural differences between them.

**Figure 6:**
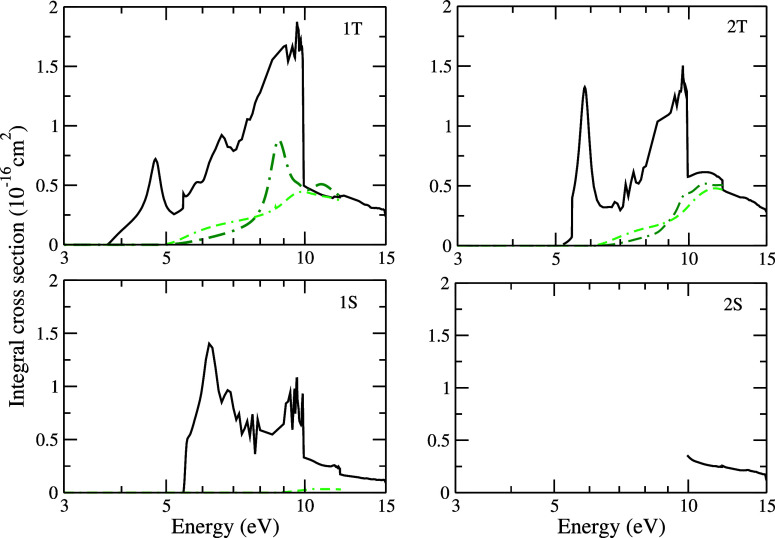
ICSs for the excitation from the ground state to the 1^3^B_2_ (3.72 eV), 1^3^A_1_ (5.11 eV), 1^1^A_2_ (5.44 eV), and 1^1^A_1_ (6.29 eV) excited states of pyrrole by electron impact. Solid black line presents SMCPP results; dashed-dotted dark green and double-dashed-dotted light green lines present results obtained by Tomer et al.^23^ using the R-matrix method with the STO-3G and DZP basis set, respectively. See the text for further discussion.

In Figure 6, integral electronically inelastic cross sections corresponding to transition from the ground state to the excited states 1^3^B_2_ (3.72 eV), 1^3^A_1_ (5.11 eV), 1^1^A_2_ (5.44 eV), and 1^1^A_1_ (6.29 eV) for electron scattering by the pyrrole molecule are presented. Hereafter, to refer to these states, we will use the acronyms: 1T, 2T, 1S, and 2S, corresponding to the first triplet, second triplet, first singlet, and second singlet states, respectively. Special attention is directed to the cross section for the transition from the ground state to the first triplet state 1^3^B_2_. At 3.74 eV, we found a structure that we believe to be associated with a resonance of mixed shape and core-excited character, which, as discussed before, is also observed in the calculation for the elastic channel. Below 10 eV, all states exhibit a significant number of structures, which may be ascribed to pseudoresonances or structures due to threshold effects. Owing to its reduced magnitude, the electronically inelastic cross sections are more sensitive to the presence of pseudoresonances or threshold effects, making it difficult to characterize the structures. At the energy of 10 eV, there is an abrupt decrease in the magnitude of the cross section, consistent with a considerable flux stealing in this region due to the increase in the number of coupled channels, going from a calculation with 5 channels to a calculation that considers 46 open channels. We compare the 1T, 2T, and 1S states obtained in our work with the results reported by Tomer et al.^23^ These authors described the excited states by using the CAS-CI method and by employing two different sets of basis functions, namely, STO-3G and DZP. The energies obtained with the more robust DZP basis set are smaller than the ones obtained with the STO-3G basis set. The threshold energies for the states provided by the authors using the different basis sets are listed in Table 5 and compared with those obtained by the MOB-SCI strategy. A significant difference is observed between the energies of the excited states, especially for the singlet excited states. Regarding the electronically inelastic cross sections depicted in Figure 6, we observe a discrepancy both in terms of behavior and magnitude compared to the results of Tomer et al.^23^ This difference can be attributed to the number of coupled channels included in the SMC and R-matrix calculations in the energy range up to 12 eV. For example, in our calculations, up to 80 open channels are considered in the energy range up to 12 eV, whereas in the authors’ calculations for the STO-3G basis set up to 7 channels are treated as open, and for the DZP basis set up to 27 channels are considered as open. The pronounced structures present in the results obtained by Tomer et al.^23^ in the 1T and 2T states are classified by them as nonphysical structures.

**Table 5:** Comparison between the Vertical Excitation Energies Obtained through the MOB-SCI Strategy with the Results Obtained by Tomer et al.^23^ Using the SAC–CI Method

state	MOB-SCI	STO-3G^23^	DZP^23^
1^3^B_2_	3.72	4.88	4.89
1^3^A_1_	5.11	6.22	6.04
1^1^A_2_	5.44	13.45	8.75
1^1^B_1_	6.29	12.45	9.66

**Figure 7:**
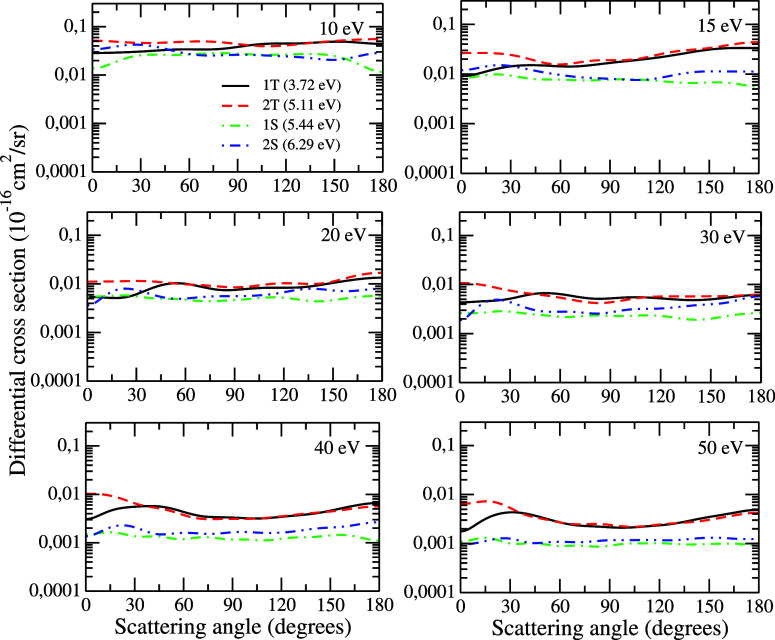
Differential cross sections for the excitation from the ground state to the 1^3^B_2_ (3.72 eV), 1^3^A_1_ (5.11 eV), 1^1^A_2_ (5.44 eV), and 1^1^A_1_ (6.29 eV) excited states of pyrrole for the impact energies of 10, 15, 20, 30, 40, and 50 eV. See the text for further discussion.

In Figure 7, the DCSs for transitions from the ground state to the first and second triplet (1^3^B_2_ and 1^3^A_1_) and singlet (1^1^A_2_ and 1^1^A_1_) states of the pyrrole molecule are presented for impact energies of 10, 15, 20, 30, 40, and 50 eV. With absolute values, typically 3 orders of magnitude smaller than elastic ones, the DCSs for the electronically inelastic processes show a pattern of oscillations that, although partially masked by the logarithmic scale, it is rich and varies with increasing energy. Furthermore, it is also possible to make some considerations about the relative magnitude of the DCSs involving the transitions to the 1T, 2T, 1S, and 2S states based on the study of Goddard III et al.,^43^ who discussed the selection rules for allowed transitions between excited states in the light of group theory. In their work, these authors highlighted that for the *C*_2*v*_ group, the transitions A_1_ → A_1_ are the most intense, followed by transitions A_1_ → B_1_ and A_1_ → B_2_, which are considered equivalent and then by transitions A_1_ → A_2_, which are the weakest. We observed the sequence ^1^A_1_ → 1^3^A_1_ > ^1^A_1_ → 1^3^B_2_ > ^1^A_1_ → 1^1^B_1_ > ^1^A_1_ → 1^1^A_2_ in the DCSs presented in this figure, which is consistent with the discussion of Goddard III et al.^43^ at energies of 10, 15, and 20 eV. For the energy of 30 eV, the ordering ^1^A_1_ → 1^3^A_1_ > ^1^A_1_ → 1^3^B_2_ is observed for angles below 30° and above 120°, while for energies of 40 and 50 eV, this same change in the ordering of the magnitude of the DCSs occurs for angles below 30° and for the angle of 90°.

## 5 Conclusions

We presented results for the elastic and electronically inelastic electron scattering by the pyrrole molecule obtained by using the SMC method within the MOB-SCI approach and considering 1–209 open channels. In the elastic channel, we identified four shape resonances, two of π* character and two of σ* character, whose signatures (character and position) are in good agreement with results previously reported in the literature.^18−23^ The present ICS curves show a similar behavior to those obtained by de Oliveira et al.,^22^ except above 8 eV, where our results have a lower magnitude due to inclusion of the electronically inelastic channels in our calculations. On the other hand, a comparison between the present DCSs with other theoretical results available in the literature displays a significant disagreement. Since no experimental data are available for electron scattering by the pyrrole molecule, we compared our results with measurements for furan, which is structurally similar to pyrrole. A very good agreement between the calculated and experimental results was observed, reinforcing the importance of considering the competition for the flux that defines the cross sections due to the inclusion of a large number of electronically excited states in scattering calculations. The excellent agreement (both in magnitude and shape) between the DCSs of pyrrole and furan, especially above 10 eV, also suggests that the continuum electron is not sensitive to the structural differences between the two systems. This is because, for energies above 10 eV, the de Broglie wavelength of the electron is on the same order of magnitude as the molecular dimensions in both systems. We also presented the ICSs and DCSs for the excitation from the ground state to the first two triplet and the first two singlet electronically excited states of pyrrole. Our computed inelastic ICSs disagree with recent theoretical results, while we have achieved excellent agreement in the elastic cross section. The DCSs for electronically excited states exhibit specific magnitude patterns that reflect the probability of excitation. These patterns are influenced by the symmetry properties of the transitions, which are governed by selection rules as used by Goddard III et al.^43^ In contrast to the elastic channel, the scenario regarding electronic excitation still poses a challenge to theory, and experiments involving the scattering of electrons by pyrrole would be very much welcomed to shed more light in the understanding of this process.
